# Integrating Care from Home to Hospital to Home: Using Participatory Design to Develop a Provincial Transitions in Care Guideline

**DOI:** 10.5334/ijic.5674

**Published:** 2022-05-20

**Authors:** Robin L. Walker, Staci Hastings, Charles Cook, Ceara T. Cunningham, Lisa Cook, Jodi Cullum, Judy Seidel, John Hagens, Scott Oddie

**Affiliations:** 1 Applied Research and Evaluation Services, Primary Health Care, Alberta Health Services, Alberta, Canada; 2 Primary Health Care Integration Network Scientific Office, Alberta Health Services, Alberta, Canada; 3 Cumming School of Medicine, Department of Community Health Sciences, University of Calgary, Calgary, Alberta, Canada; 4 Faculty of Health Sciences, University of Lethbridge, Lethbridge, Alberta, Canada; 5 Faculty of Social Sciences, University of Calgary, Calgary, Alberta, Canada

**Keywords:** guideline, integration, participatory design, transitions in care

## Abstract

**Introduction:**

Patients worldwide experience fragmented and uncoordinated care as they transition between primary and acute care. To improve system integration and outcomes for patients, in 2017/2018 Alberta Health Services (largest health services delivery organization in Canada) called for a coordinated approach to improve transitions in care (TiC). Healthcare leadership responded by initiating the development of a province-wide guideline outlining core components of effective transitions in care. This case study highlights the extensive design process used to develop this guideline, with a focus on the participatory design (PD) approach used throughout.

**Methods:**

An iterative, mixed methods PD approach was used to engage over 750 stakeholders through the following activities to establish Guideline content: i) learning collaborative; ii) design-team; iii) targeted online surveys; iv) primary care stakeholder consultation; v) modified Delphi panel; and vi) patient advisory committee.

**Results:**

The result was Alberta’s first guideline for supporting patients through TiC: “Alberta’s Home to Hospital to Home Transitions Guideline”.

**Conclusion:**

The extensive design process used to create the Guideline was instrumental in establishing content, encouraging system integration, and creating conditions to support provincial implementation. While intended to improve and standardize patient care in Alberta, the methods used and lessons learned throughout the development of the Guideline are applicable internationally.

## Introduction

Outcomes of poor transitions in care (TiC) (sets of actions designed to ensure safe and effective coordination and continuity of care as patients change providers, locations, or health status) [1] are multifactorial and well documented [2,3]. Poor transitions result in high rates of avoidable readmissions to hospital or visits to the emergency department, accelerated progression of disease, early mortality, reduced quality of life, and care misaligned with patient preferences [4,5,6,7]. While health service delivery organizations globally have made limited progress in improving TiC [8,9,10,11], efforts have been made to address TiC gaps in the province of Alberta, Canada.

Alberta Health Services (AHS) is the single largest health delivery organization in Canada, providing health services across five geographical zones to 4.4 million people through universal healthcare coverage [12]. While AHS focuses primarily on acute care delivery, primary care is provided predominantly by independent providers in community [13]. As a result, integration between primary and acute care is a significant challenge [14,15] and ~30% of adult Albertans experience a significant gap in care during transitions from hospital to home (e.g., inadequate follow-up or information provided at discharge; care plans not shared across settings and providers) [2,3,16].

As a first point of contact and a reliable medical resource to communities they serve, primary care providers (PCPs) are at the heart of the healthcare system, caring for and supporting patients throughout all interactions with the healthcare system. PCPs in Alberta consistently report a lack of integration with AHS’s acute care system and the impact it has on their ability to provide timely, comprehensive care for patients transitioning from hospital back home [17]. The need to shift the view of TiC from a hospital-centric set of interventions, such as focusing solely on discharge processes, toward new approaches involving the entire care continuum (including PCPs; community providers; and patients, families and caregivers) is evident [12].

In 2017/18, the Government of Alberta called for a coordinated approach to improve patient transitions [18]. Healthcare leadership in Alberta responded by initiating the development of a province-wide guideline providing operational areas of the healthcare system guidance on key components required to achieve effective TiC. Transitions between hospital and home are challenging, complex, multi-step processes requiring integrated communication and coordination across patients, caregivers, and providers in primary, acute and community care [19]. The intent of this guideline was to bridge these key connections between hospitals, primary care, and community services, with patients, families and caregivers at the center. Given the complexities of developing and implementing a TiC guideline that spans across primary and acute care, an extensive participatory design (PD) process involving a multitude of key stakeholders was needed.

Although the theoretical underpinnings and processes of PD have evolved since emerging in the 1970’s [20,21], an “infrastructuring” PD approach was deemed most appropriate for the development of the Guideline. This approach is conceptualized as designing for future use [21], or design-after-design [20], wherein the PD process creates conditions for sustained, ongoing design among participants after design in a specific project [20,21,22]. Infrastructuring acknowledges that design and social innovation involving groups of heterogeneous stakeholders is more often characterized by controversy than consensus, emphasizing the importance of enabling user adoption and appropriation beyond the initial scope of design [20,21]. This PD approach is guided by two sets of values: the value of democracy and the value of tacit knowledge [20]. While the value of democracy stresses the importance of proper, legitimate user participation, the value of tacit knowledge affirms the importance of participants’ practical and informal skills in the design process [20].

The shortcomings of traditional project design approaches that progress through consecutive stages of refinement (i.e., analysis, design, construction and implementation) are well-documented [20]. Such shortcomings include top-down methods of design that hinder adaptation to changing conditions, rigidity of specifications, and hierarchical structures averting legitimate participation [20]. Considering the conditions needed to design and implement a provincial guideline promoting integration across the health system, these limitations highlight the inappropriateness of using traditional approaches to project design for this purpose. For reasons explained subsequently, a PD approach was chosen to develop the Guideline.

In general, PD is rooted in the simple notion that future users affected by a design should be involved in the design process [20,23,24,25]. PD is an iterative process in which knowledge, experience, and perspectives from a broad range of stakeholders are collected to create shared understanding and reach decisions in areas of scientific uncertainty or disagreement [25,26,27]. PD encourages stakeholders to work side-by-side throughout the design process to ensure results are appropriate and responsive to the needs of end-users [23,25]. Moreover, PD processes are guided by democracy [23,24], equalized power relations [24], trust [25], genuine shared decision making [24,26,27], bi-directional communication [4,8], tacit knowledge [26,28], and stakeholders learning both *with* and *from* one another [23,25,27].

Benefits of PD are well-documented in the literature. Since emerging approximately 30 years ago [29], recognition of the benefits of PD in improving healthcare has increased globally and efforts to augment stakeholder and public engagement in healthcare redesign has continually grown [30,31,32]. PD empowers stakeholders by promoting collaboration, moderating power differences [25,28,33], and converting stakeholder suggestions into tangible outcomes [28]. Importantly, PD positively impacts implementation and fidelity of results [24,28]. Products or services designed using PD are often more appropriate and robust than those designed through non-PD methods [23]. Involving stakeholders who understand the context in which newly designed products will be used leads to higher acceptance and greater likelihood of uptake and sustained use [23,28,34]. Together, these principles are inherently favorable to the design, development and implementation of a new provincial guideline.

Although not new to healthcare [28], the principles of PD have not traditionally been applied in healthcare settings to their fullest extent. Stakeholder participation in healthcare improvement activities has traditionally been characterized by unequal distributions of power, where patients and community stakeholders hold passive roles relative to healthcare providers and leadership [27,30,35]. Progress to involve stakeholders to a greater extent in healthcare improvement activities has also been slow and often consistent with lower levels of engagement [36]. Literature shows few healthcare organizations mention empowerment when describing their engagement strategies [36] and patients are primarily engaged via consultation activities [37]. To address power imbalances and endorse full stakeholder participation in healthcare improvement efforts, broad democratic models of engagement within health organizations are required [36].

The case study presented in this paper demonstrates the application of multiple methods used to create Alberta’s first provincial TiC Guideline, with a particular focus on infrastructuring PD [20,21,22,38]. The research team aimed to address research-practice gaps and truly impact TiC in Alberta through processes encouraging stakeholders to co-create alongside one another, fostering working relations that allow for development of continuous partnerships and different design possibilities to be explored after completion of the Guideline.

## Guideline design process

**Figure 1:**
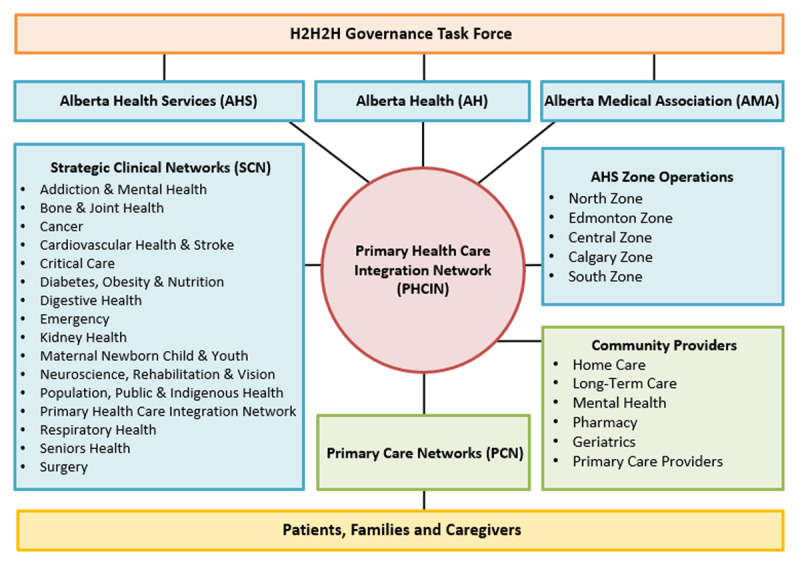
Stakeholder groups involved in the design of Alberta’s Home to Hospital to Home (H2H2H) Transitions Guideline.

From April 2018 to November 2019, more than 750 diverse stakeholders were involved in an extensive design process to design content for the Guideline (see Figure 1).

**Figure 2:**
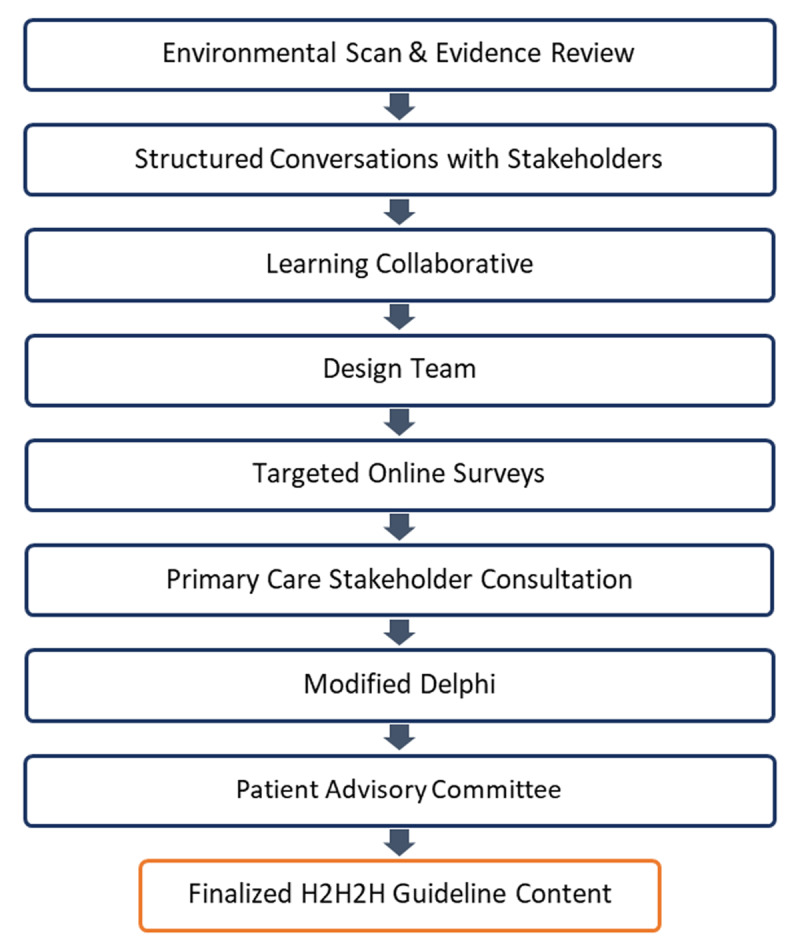
Overview of the design process used to develop Alberta’s Home to Hospital to Home (H2H2H) Transitions Guideline.

The research team utilized iterative mixed methods, where stakeholder perspectives from one phase informed the next. The first stage of Guideline content development included an environmental scan, evidence review [39], and structured conversations with key stakeholders. This led to an initial draft containing 8 themes which constituted a quality TiC from home to hospital to home and served as the foundation for the remaining design process: the focus of this case study. Following this first draft, key stakeholders were continually engaged, multiple iterations of the Guideline were produced, and Guideline content was finalized through the following methods: i) learning collaborative; ii) design-team; iii) targeted online surveys; iv) primary care stakeholder consultation; v) modified Delphi panel; and vi) patient advisory committee (see Figure 2). The Guideline was revised throughout the entire design process to reflect the results of each stage.

### i) Learning Collaborative

Learning Collaboratives, or “co-labs”, are an innovative method designed to find solutions to healthcare challenges such as TiC [40]. In December 2018, 53 stakeholders from across Alberta attended a full day co-lab meeting to inform them of Guideline work to date, capture their perspectives on TiC, and collect feedback to begin refining Guideline content. In attendance were: patients and families, AHS zone operations, Alberta Medical Association (AMA) [41], community and healthcare providers (e.g., acute care, primary care, homecare, pharmacy, emergency medical services, long term care), Strategic Clinical Networks, and Alberta Health.

During the meeting, stakeholders rotated through six stations coinciding with one or two elements of the first iteration of the Guideline. Two facilitators guided discussions at each station to identify key points within each element and capture stakeholder experiences of what constitutes a quality TiC (e.g., processes). For example, stakeholders were asked to “imagine you are a patient and/or provider and share with us what things you need in place to achieve safe transitions”. These conversations were documented and later themed using a content analysis approach [42] to identify points of consensus within each Guideline element. Results were shared back and validated with stakeholders before making amendments to the Guideline content.

#### Results

Theming indicated stakeholders reached consensus on many areas across Guideline elements. Examples included clarifying key roles and responsibilities/accountabilities throughout the transition process and leveraging existing provincial infrastructure and activities to achieve effective TiC (e.g., committees, processes, initiatives, tools, IT systems, etc.). Stakeholder feedback also resulted in a reduction from eight Guideline elements down to six. In addition to identifying where consensus was reached, the research team identified content stakeholders did not reach consensus on. To clarify that content, questions were formulated to guide discussion at the subsequent stage of the Guideline design process. Examples of questions taken forward were: what are the minimum specifications for the information included in a discharge summary (i.e., what standard information should be included) and who is responsible for a patient at each stage of their care? Stakeholders strongly recommended the research team form a diverse design team to address topics of non-consensus and further develop Guideline content.

### ii) Design Team

Approximately 70 design team members, including patient advisors, physicians, and allied health professionals, contributed their perspectives during this phase of Guideline development. Some members from the preceding co-lab meeting volunteered to be part of the design team, while most were recruited by the research team to ensure a wide range of representation. Design team members had the choice to attend an in-person focus group in March 2019 or one of two virtual focus groups hosted in April 2019. During each focus group, participants were divided into three groups and assigned questions to clarify Guideline content. For example, stakeholders were asked “what should be in scope for how to coordinate care between hospital and community?” All focus groups were recorded (approximately 15 hours of group discussion was captured), transcribed, and analyzed [42] to identify key themes across design team member responses.

#### Results

Content was added to the appropriate elements of the Guideline reflecting the key themes identified. The addition of patient attachment [43] and outlining how PCPs should be involved in receiving, reviewing, and acting upon transition notifications are examples of changes made to Guideline content as a result of the design team focus groups. Analysis also highlighted where lack of agreement/consensus remained. For instance, title changes were recommended for four of six Guideline elements and questions were raised regarding recommended timeframes for sending admit notifications. As advised by design team members, these questions and all other unresolved content was brought forward and discussed through targeted group consultations.

### iv) Targeted Online Surveys

To strengthen stakeholder ownership of the Guideline, collect perspectives to inform an implementation strategy, and continue refining Guideline content, stakeholders were consulted through targeted online surveys. Surveys were designed for four strategically chosen key stakeholder groups: 1) AHS Zone Operations, 2) Strategic Clinical Networks (SCNs), 3) Primary Care Networks (PCNs), and 4) PCPs. AHS Zone Operations, including managers and administrative leads from acute care, home care, and many other areas, were engaged given their accountability to oversee implementation of the completed Guideline within their zones across the province. Due to their roles transferring evidence to practice, coordinating collaboration across institutional and organizational boundaries [44], and reaching out to a broad range of acute care stakeholders, SCN stakeholder engagement was key. PCNs, which are networks of physicians and other healthcare providers that deliver primary healthcare services and programs to Albertan’s [45], were targeted to gather their expertise in coordinating access and follow-up to primary healthcare. Lastly, PCPs were targeted not only for their clinical expertise, but also to garner buy-in and collect direction from providers accountable for implementing best practices, processes, and tools included in the Guideline. Each survey was designed with questions specific to the expertise and experiences of each key stakeholder group. For example, PCP survey questions targeted terminology/language and content related specifically to primary care (e.g., minimum specifications for a hospital admission notification). A pilot study (n = 10) was conducted with stakeholders to assess face validity in each questionnaire. Surveys were created using Simple Survey^TM^ and distributed through the research team to key representatives of each stakeholder group in June 2019. Respondents were surveyed utilizing snowball sampling. Each group had 14 days to complete the survey from time of initial distribution. Participants were not incentivized to participate and received a series of reminders.

#### Results

There were 296 respondents to the online surveys (AHS Zone Operations (n = 99), SCNs (n = 39), PCNs (n = 42) and PCPs (n = 116)). Finite mixture modelling and ordinal logistic regression were used to determine disagreement within and between groups, respectively. The results from these surveys were used to revise and update Guideline content where agreement was reached. For example, majority of stakeholders in all four groups agreed to incorporate the Provider Discharge Summary element into the Transition (Discharge) Planning Process element. Two examples of outstanding questions included “to what extent do you agree the leading operational practice for primary care providers should be “all patients discharged from the hospital should be contacted by their primary care office via phone, email, or text, etc. within one week of discharge”?” and “If a patient leaves hospital without the recommended primary care appointment booked, whose responsibility is it to book this appointment to ensure it occurs within the recommended time frame?” To continue developing content and reaching consensus, consultations with PCPs were completed.

### v) Extended Primary Care Stakeholder Consultation

From the initial literature review and the outputs of the online PCPs survey, it was evident there was a lack of evidence related to what constitutes an effective primary care follow-up post hospital discharge [46,47,48]. Given this gap, tacit knowledge specifically from family physicians was required. This strategy was also an implementation tactic given primary care is not part of the larger AHS system, but rather a joint venture partner. With primary care positioned external to AHS as a service delivery partner, engaging and incorporating the perspectives of PCPs was important to create an integrated guideline and increase likelihood of future implementation in primary care.

Fourteen PCPs were recruited through the AMA. Each PCP completed a one-hour interview, followed by a three-hour focus group. Interview questions focused on processes; roles and responsibilities of acute, primary and community care; informational and management continuity; potential gaps in Guideline content; and patient profiles. For example, PCPs were asked “what supports (tools, resources and information) are needed for an effective initial follow-up visit on transition from hospital?” The interviews and focus group were recorded, transcribed, and themed [42] to identify areas of consensus/non-consensus.

#### Results

Themed results revealed agreement among PCPs that acute care should be responsible for organizing recommended specialty follow-up, including procedures, diagnostic imaging, and referrals that need to occur post-hospitalization. Another example of consensus reached was to reinforce within the Guideline that patients will leave hospital with a transition plan that includes all required education materials they can take to their follow-up appointment with their PCP in primary care. Additional content suggestions that materialized from PCP consultations to be discussed during future design activities included: timing and content of a discharge summary, prescribing sufficient medication for patients until post-discharge follow-up with primary care is required (i.e., opioids); and what should PCPs/teams do if things do not go as planned (i.e. tests/procedures scheduled by the specialist did not occur)? The research team incorporated content PCPs agreed upon into the Guideline prior to entering the last phase of Guideline development: a Modified Delphi panel.

### vi) Modified Delphi Panel

A modified Delphi panel process [49,50,51], effective for engagement and reaching consensus [52], was used to finalize Guideline content where consensus had not yet been reached among key stakeholders. Content discussed during this phase of the design process came from previous stakeholder consultations. Panelists were carefully considered and included 28 provincial providers and leaders involved in TiC across a variety of disciplines (e.g., acute care, primary care, community care, pharmacy, etc.) who had not previously been involved in the Guideline design.

**Figure 3:**
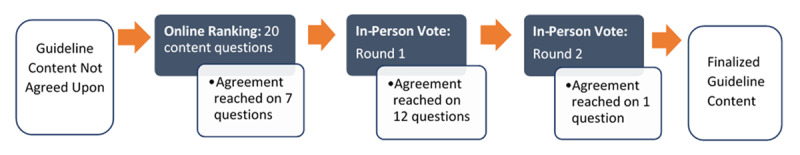
Modified Delphi panel review process and results.

Panelists were provided a summary of the project and an overview of the consensus process (e.g., time commitment and activities) prior to formally agreeing to participate. The panel review process involved reviewing the draft Guideline, an online ranking process, and a full-day face-to-face meeting (see Figure 3). A modification of the RAND appropriateness rating methodology was used to rate materials on a 10-point scale. This 2-step rating process [53] (i.e., initial ratings done in isolation followed by face-to-face discussion) is recognized as highly appropriate for this type of content judgment. Drawing upon RAND definitions of agreement [54], panel agreement was achieved during the online ranking process when median scores were >7.5 (include content) and <5 (exclude content). Content without such agreement was discussed at the face-to-face meeting and voted upon. The types of questions/content presented to panel members included: “what are the key actions that patients, caregivers and families are accountable for?” and “should primary care be responsible for arranging the follow-up appointment with a specialist?” Up to three rounds of voting can occur during a Modified Delphi panel and consensus is reached with a majority vote of ≥75%. If consensus is not reached in three votes, a final decision is based on majority vote.

#### Results

Of 20 content questions presented to panel members during the online ranking stage of the Delphi process, agreement was reached on seven of them. The remaining 13 questions were taken forward to the face-to-face meeting to be discussed and voted on. As shown in Figure 3, panel members reached consensus on all 13 questions in 2 rounds of voting or less. An example of content agreed upon and included in the Guideline was “in the Admit Notification component of the Guideline, patients, families and/or caregivers should be responsible for providing hospital staff with an updated medication list (including herbal medications, supplements, and other non-prescription medications).” The results from the Delphi process overall were used to make final revisions to the Guideline before seeking endorsement from provincial leadership (e.g., Provincial Primary Care Network Committee and AHS Joint Venture Council).

### v) Patient Advisory Committee

A Patient Transitions Resources (PTR) team comprised of four patient and family advisors and 3 AHS Primary Health Care staff members were key to the development of the Guideline. The PTR team brought the patient-voice forward to ensure what patients and families need for safe, patient-centered TiC journeys was considered throughout the design process. The PTR Team also created a patient-oriented companion guide that accompanies the Guideline, containing six recommendations for health system leaders and resources patients and families need for effective, patient-centered transitions (available at https://albertahealthservices.ca/assets/info/hp/phc/if-hp-phc-phcin-hthth-patient-report.pdf). An additional small group of patients/family/caregiver advisors (10–15 attendees) worked with the PTR Team and participated in three webinars to create content relevant to the “roles and accountabilities of patients, families and caregivers” within the Guideline.

Engaging patients throughout the development of both the Guideline and companion guide was critical to ensuring both resources were relevant and useable among patients and caregivers (not just healthcare providers) and achieving integration at a system level.

## Results of the extensive participatory process

**Figure 4:**
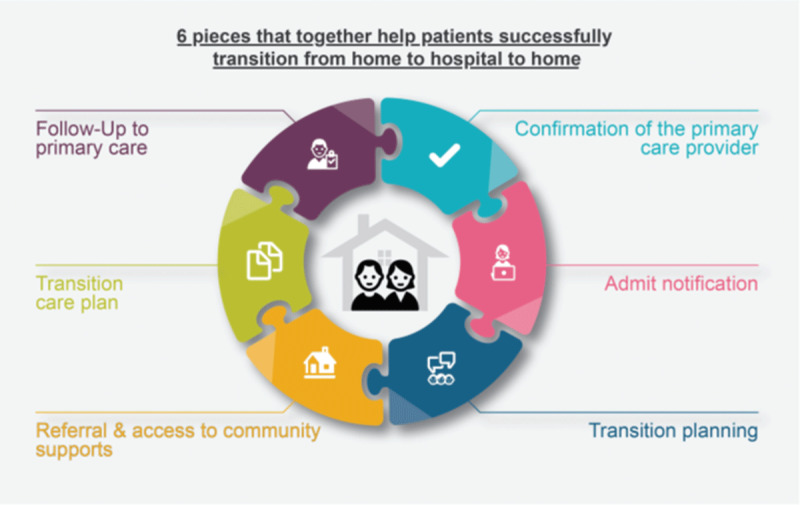
The six elements of Alberta’s Home to Hospital to Home Transitions Guideline.

Results of the PD process and stakeholder involvement in each of the design phases described above was Alberta’s first provincial guideline for supporting adult patients as they transition from community, to hospital, and back home: Alberta’s Home to Hospital to Home (H2H2H) Transitions Guideline (publicly available at www.ahs.ca/hhhguideline). This guideline bridges connections between hospitals, primary care, and community services, with patients, families and caregivers at the center. The final version is comprised of 6 elements (see Figure 4), each divided into leading operational practices for hospital team; PCPs/team; patients, families, and caregivers; and community supportive care team(s); tools and resources; change management tips; and additional information. Each section is foundational in the patient journey, building upon one another to facilitate high quality transitions and enhance integration across the health system.

## Discussion

Over a year and a half, providers from primary care, acute care and community united alongside patients, researchers, government, and healthcare operations leaders to co-design a provincial guideline to support patients as they transition from community, into hospital, and back home again. The H2H2H Guideline was developed using an extensive PD process with input from over 750 stakeholders. This approach was instrumental in developing content that could not be informed solely through existing literature developing processes and communication streams to improve integration across the Alberta health system. The PD approach was anchored in the continuous design after design concept [20], where design at use provides ongoing infrastructuring to support implementation and future evolution. In other words, the Guideline spans across space and time and will evolve to include stakeholders post-design as it is rolled out across Alberta to achieve broad system change and shift culture around transitions in care.

One of the key challenges faced during this PD process was the dynamic tension between using an evidence-based approach (i.e., science, rigor and established expert contributions) versus tapping into tacit knowledge of designers. This tension is well documented in PD approaches [20] and knowledge mobilization literature [28]. Bjorgvisson et al. [20] addresses this challenge, highlighting the importance of using participants’ tacit knowledge in the design process – practical and diverse skills fundamental to the making of the design object (i.e., Guideline) – not just formal and explicit competencies. Greenhalgh et al. [38] and Langley et al. [28] describe this as “collective sense making”, defined as “the collaborative generation of knowledge by academics working alongside stakeholders from other sectors” [38, p. 393]. Part of the knowledge mobilization process includes moving beyond the notion of pure academic, scientific, and empirical evidence, towards dynamic and adaptive community-academic partnerships co-creating knowledge [38]. For example, in our case study, limited evidence is currently available regarding the value of having a patient’s care team (e.g., PCP) involved early in the transition process [55]. However, stakeholders were adamant about the importance of involving a patient’s circle of care early in the transition process as it allows for bi-directional flow of information between primary and acute care; an opportunity for a patient’s circle of care to contact the hospital team (e.g., attending physician) to convey vital information (e.g., patient history, care plan, medications). Referring to stakeholders to fill gaps where the evidence was limited helped achieve role clarity, cross buy-in, and broad consensus across stakeholders and effectively address tension between the use of science versus tacit knowledge that was evident in our study.

In Alberta and across Canada, there is a long-standing history of tension between primary care and the acute care system [56]. It is well “documented” that primary care is the foundation of an effective health system when integrated with acute and specialty care [57]; however, multiple structures, governance and funding models currently in Alberta introduce challenges that require specific attention to achieve system integration. Thus, developing a guideline for TiC that spans acute, primary and community care poses an opportunity to improve system integration. Politically, it was a challenge getting stakeholders at the proverbial table given the inherent tensions between our stakeholder groups. The politics of `emotional engagement’ vs. `evidence-based reflections’ during our design discussions were always present. Consequently, balancing representation and stakeholder opinion was difficult throughout this PD process. Nevertheless, using a PD approach provided a unique opportunity for designers to hear new ideas, perspectives, and alternative solutions from other stakeholders and jointly collaborate in designing standardized recommended care and transition follow-up processes. Examples of this include: 1) developing mechanisms/processes for primary care to be involved in transitions upon hospital admission; 2) acute care having responsibilities post-hospital discharge and; 3) identifying the responsibilities of patients in their own care. Ultimately, stakeholders came to the table with a single purpose: to determine what patients’ truly need for successful transitions.

A final key design challenge this team encountered was to design in the absence of consensus. This is a common challenge identified in PD literature [20,58,59]. The public is characterized by heterogeneity and conflict; thus, designing for, by, and with stakeholders can be challenging and create conflict and disagreement across stakeholder groups where common objectives and solutions already exist [58,59]. As mentioned above, there was inherent heterogeneity and differences across groups of stakeholders in the current case study. To overcome this challenge, the study team supported partners in exploring the applicability of new ideas, visions, and possible solutions to their own local context. This is in line with an “infrastructuring” approach to design: allowing for continuous design such as changes and adaptations to accommodate regional or local differences. This was considered a key principle for implementation of the Guideline (i.e., allow for adjustments when and where needed).

Other factors vital to implementation success include a common language [60], shared understanding of the rationale for the work [60], and shared ownership [23]. A common language, free of semantic nuance, is required to achieve long-lasting change in health service delivery [60]. A major focus of stakeholders during the development of the H2H2H Guideline was shifting the language and terminology used within the Guideline to be more reflective of the processes and aims of successful patient transitions. For example, stakeholders came to agreement on changing what has been traditionally termed the “discharge summary” to a “transition care plan” to reflect how discharge is only one piece of the transition journey (i.e., transition is a more appropriate term given it emphasizes a journey beyond the hospital walls). The PD methods used in this case study were also successful in communicating the purpose, or rationale, for developing this TiC Guideline. The research team dedicated time at the start of every participatory activity to reiterate the source of the Guideline mandate, describe TiC gaps prominent in the literature, highlight patient stories, and communicate the goals and desired impact of the Guideline on patient outcomes. Much conversation among stakeholders during each participatory phase also centered on sharing their experiences and anecdotal evidence of poor transitions, all of which reinforced the importance of the work. Lastly, by incorporating the perspectives, expert opinions, and lived experiences of stakeholders into the Guideline at each stage of the design process and validating back with stakeholders prior to subsequent design phases, participants witnessed and could identify their contributions to the Guideline, creating a sense of shared ownership across all involved.

The research team identified many facilitators and barriers through their experiences leading a large, diverse group of stakeholders through the extensive PD process used to create the H2H2H Guideline. Although this process took place in the province of Alberta, the lessons learned are applicable internationally.

## Lessons learned

- An important enabler for this project was clear, effective, organizational leadership from within AHS. Several empirical studies have highlighted the significant role of leadership involvement in achieving successful collaboration and integration [61,62].
- Using an applied, participatory approach (versus a traditional academic approach) to develop a provincial guideline aimed at solving a practical problem in the healthcare system was very successful. This approach allowed for: flexibility in methods (i.e., stakeholders influenced types of methods used), important consideration of clinical experience, and an end-product that can be revised and updated alongside the changing health system.
- PD methods enhanced communication and fostered relationship building among stakeholders across the entire design process, creating an opportunity for stakeholders to gain a detailed understanding of each other’s role in the transition process.
- Providing a shared space for traditionally divergent groups such as acute care, primary care, AHS operations, and patients to work collaboratively was identified as an important enabler to achieving integrated care models.
- PD methods used throughout the design process were critical to creating a sense of ownership among stakeholders and ensuring the Guideline met end-user needs: key to successful implementation.

## Conclusion

An extensive design process was successfully used to design a TiC Guideline that crosses multiple sectors across the healthcare system (i.e., acute, primary, and community care). This project used a participatory approach to develop Alberta’s H2H2H Transitions Guideline to address all points in a patient’s transition journey, reinforce a patient-centered provincial approach to TiC, and identify responsibilities of care providers involved at each transition point. The extensive design process in this case study was used to reach consensus and shared understanding, reflect diverse stakeholder content needs to achieve integration, and create conditions favorable for successful implementation across the province. Key challenges of this approach include 1) balancing the dynamic tension between using an evidence-based approach (i.e., science, rigor and expertise) versus tapping into tacit knowledge, 2) balancing representation and stakeholder opinion and, 3) to design when reaching consensus is difficult. Valuing tacit knowledge as expert opinion, embracing conflict and allowing room for local adaptation are possible strategies teams can use to overcome these challenges. Ultimately, this innovative approach to coordinating transitions across the continuum of care was intended to improve integration, system effectiveness, and continuity of care between acute, primary, and community care.
